# Gene expression analysis in asthma using a targeted multiplex array

**DOI:** 10.1186/s12890-017-0545-9

**Published:** 2017-12-11

**Authors:** Christopher D. Pascoe, Ma’en Obeidat, Bryna A. Arsenault, Yunlong Nie, Stephanie Warner, Dorota Stefanowicz, Samuel J. Wadsworth, Jeremy A. Hirota, S. Jasemine Yang, Delbert R. Dorscheid, Chris Carlsten, Tillie L. Hackett, Chun Y. Seow, Peter D. Paré

**Affiliations:** 1 UBC Institute for Heart Lung Health, St. Paul’s Hospital, 1081 Burrard St, Vancouver, BC Canada; 2 UBC Department of Medicine, Division of Respirology, University of British Columbia, Vancouver, BC Canada; 3 UBC Chan-Yeung Centre for Occupational and Environmental Respiratory Disease, Gordon & Leslie Diamond Health Care Centre, Vancouver General Hospital, 2775 Laurel Street, 7th floor, Vancouver, BC Canada; 4 University of British Columbia Centre for Heart Lung Innovation, St. Paul’s Hospital, 1081 Burrard St, Vancouver, BC Canada; 5 UBC School of Population and Public Health, University of British Columbia, Vancouver, BC Canada; 6 UBC Department of Anesthesiology, Pharmacology and Therapeutics, University of British Columbia, Vancouver, BC Canada; 7 UBC Department of Pathology and Laboratory Medicine, University of British Columbia, Vancouver, BC Canada; 8 Division of Respirology, Department of Medicine, McMaster University, Hamilton, ON Canada; 9 Children’s Hospital Research Institute of Manitoba, 513-715 McDermot Avenue, Winnipeg, MB R3E 3P4 Canada

**Keywords:** Asthma, Co-expression, Nanostring, Extracellular matrix, CTCF, Smooth muscle, Remodeling, Epithelium, Targeted expression

## Abstract

**Background:**

Gene expression changes in the structural cells of the airways are thought to play a role in the development of asthma and airway hyperresponsiveness. This includes changes to smooth muscle contractile machinery and epithelial barrier integrity genes. We used a targeted gene expression arrays to identify changes in the expression and co-expression of genes important in asthma pathology.

**Methods:**

RNA was isolated from the airways of donor lungs from 12 patients with asthma (8 fatal) and 12 non-asthmatics controls and analyzed using a multiplexed, hypothesis-directed platform to detect differences in gene expression. Genes were grouped according to their role in airway dysfunction: airway smooth muscle contraction, cytoskeleton structure and regulation, epithelial barrier function, innate and adaptive immunity, fibrosis and remodeling, and epigenetics.

**Results:**

Differential gene expression and gene co-expression analyses were used to identify disease associated changes in the airways of asthmatics. There was significantly decreased abundance of integrin beta 6 and Ras-Related C3 Botulinum Toxin Substrate 1 (RAC1) in the airways of asthmatics, genes which are known to play an important role in barrier function. Significantly elevated levels of Collagen Type 1 Alpha 1 (COL1A1) and COL3A1 which have been shown to modulate cell proliferation and inflammation, were found in asthmatic airways. Additionally, we identified patterns of differentially co-expressed genes related to pathways involved in virus recognition and regulation of interferon production. 7 of 8 pairs of differentially co-expressed genes were found to contain CCCTC-binding factor (CTCF) motifs in their upstream promoters.

**Conclusions:**

Changes in the abundance of genes involved in cell-cell and cell-matrix interactions could play an important role in regulating inflammation and remodeling in asthma. Additionally, our results suggest that alterations to the binding site of the transcriptional regulator CTCF could drive changes in gene expression in asthmatic airways. Several asthma susceptibility loci are known to contain CTCF motifs and so understanding the role of this transcription factor may expand our understanding of asthma pathophysiology and therapeutic options.

**Electronic supplementary material:**

The online version of this article (10.1186/s12890-017-0545-9) contains supplementary material, which is available to authorized users.

## Background

Asthma is a chronic inflammatory disease of the airways, characterized by symptoms of breathlessness, wheezing and cough, associated with variable airflow limitation and airway hyperresponsiveness (AHR). Asthma is also characterized by airway remodeling which includes goblet cell metaplasia, epithelial damage, subepithelial fibrosis, basement membrane thickening, and increased airway smooth muscle (ASM) mass [1]. The pathogenesis of asthma is believed to involve an interaction between the innate and adaptive immune systems [2], and phenotypic changes within the epithelial-mesenchymal trophic unit [3]. Genetic and genomic analyses have been used to discover the molecular mechanisms underlying these phenotypic changes. Large genome-wide association studies (GWAS) have reproducibly identified single nucleotide polymorphisms (SNPs) in or near genes predominantly expressed in the airway epithelium and immune cells as susceptibility factors for asthma. The specific genes include gasdermin B (*GSDMB*) [4], interleukin 33 (*IL33*), and thymic stromal lymphopoietin (*TSLP*) [5]. Another candidate gene *ADAM33*, expressed in ASM, has been shown to be associated with asthma in linkage analysis [6]. Genetic variants associated with susceptibility for asthma may exert their effect by altering gene expression levels; indeed many of the SNPs associated with asthma and AHR have been shown to be expression quantitative trait loci (eQTL) in lung tissue, epithelial and blood cells, and altered protein expression of some of these genes has been found in cells and tissue from asthmatic individuals [7].

In this hypothesis driven study we used a reproducible multiplexed technology (Nanostring®) to quantify the expression of 334 genes potentially involved in phenotypic changes in asthmatic airways. This technology is highly sensitive, reproducible, and is suitable for archived tissue specimens as it is insensitive to RNA degradation [8]. We hypothesized that changes in the expression patterns of genes involved in ASM contraction, the cytoskeleton, epithelial barrier function, innate/adaptive immunity, fibrosis and remodeling, and epigenetics would be present in the airway tissue of asthmatics compared to non-asthmatics. Our results suggest that alterations in the expression of genes involved in cell-cell and cell-matrix interactions may contribute to the pathogenesis of asthma, particularly severe asthma. The identification of altered gene co-expression networks may identify changes in transcriptional regulation that could be pathogenic and missed with commonly used analyses for differential expression.

## Methods

Additional description of methods is provided in the online supplement.

### Subject selection and RNA isolation

**Fig. 1:**
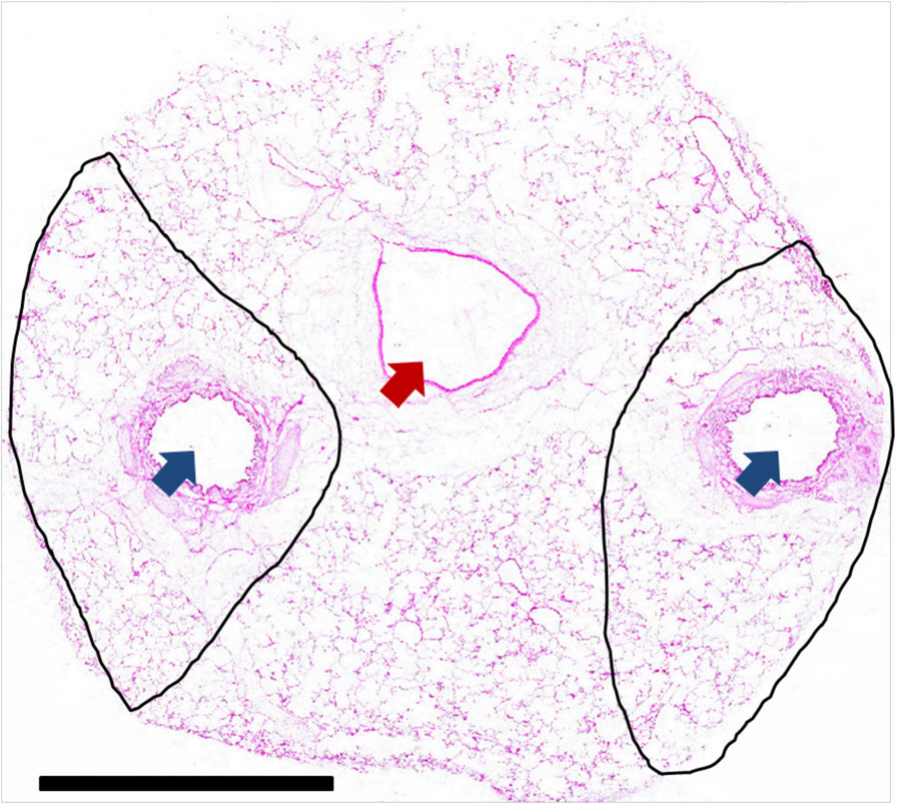
Section of a frozen human lung core cut at 10 μm thickness. Blue arrows indicate airways. Red arrow indicates a blood vessel. The black lines show the outline of the tissue taken for isolation of total airway RNA. Scale bar = 5 mm

Human lungs were donated with consent from the IIAM and used with approval from the University of British Columbia and St. Paul’s Hospital ethics committee. Diagnosis of asthma was determined through patient medical history and asthma medication usage as determined by family interview. Non-asthma donor deaths were primarily due to head trauma while 8 of the 12 donors with asthma died during exacerbations of their asthma. The other four donors with asthma died due to other, accidental causes (eg. head trauma). Subject demographics can be seen in Table 1 with full subject characteristics found in Additional file 1: Table S1. After surgical removal the lungs were flushed with Custodiol HTK solution (Odyssey Pharmaceuticals, East Hanover, NJ, USA) and transported on ice by plane. The time between harvesting and arrival at the University of British Columbia was 15–20 h. Tissues from the lungs have been used in previous studies [9–11]. Inflated frozen lungs were processed into tissue cores for sectioning on a crytostat. A total of twenty 10 μm thick sections per core were cut and stored at −80 °C until RNA was isolated. Sections 1, 5, 10, 15 and 20 were stained with hematoxylin and eosin (H&E) for morphometric measurements. For the remaining 15 sections, airways and a small amount of surrounding parenchyma were macroscopically dissected using a scalpel (Fisher Scientific® No. 11) for RNA isolation. Samples were only used if the airways seen on the first section were continuous for the 20 sequential sections. Large vessels were avoided. Sample airway is seen in Fig. 1. RNA was isolated using the Qiagen® RNeasy Mini Kit according to manufacturers protocol.

**Table 1:** Patient demographics. Ages not significantly different

	Non-asthmatic (*n* = 12)	Asthmatic (n = 12)
Median Age (Range)	21 (4–63)	17.5 (8–36)
Male Sex – # (%)	6 (50)	7 (58.3)
Average Weight - kg (±SEM)	75.7 (7.5)	68.8 (6.4)
Inhaled Corticosteroids – # (%)	0 (0)	6 (50)
Smoking - # (%)	2 (16.7)	4 (33.3)
End of life steroids - # (%)	4 (33.3)	8 (66.7)
Fatal Asthma - # (%)		8 (66.7)

### Characterization of airway dimensions

**Fig. 2:**
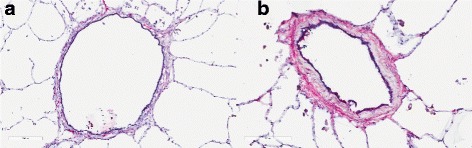
Comparison of non-asthmatic airway (**a**) and asthmatic airway (**b**). Airways are stained using hematoxylin and eosin (H&E) to highlight remodeling changes within the airways. Scale bar is 200 μm

Measurements of ASM, epithelial, collagen, and total wall area in addition to basement membrane thickness were measured to quantify the degree of airway remodeling. The five sections stained with H&E (Fig. 2) from each core were digitally scanned and airway wall compartments were quantified using the Aperio® system (Leica Biosystems, Germany). ASM area, epithelial area and total airway wall area were quantified using a point counting method, where a grid of 4000 points was overlaid onto each airway of interest and the points falling on the area of interest were counted (Image Pro Plus ®, Media Cybernetics, Maryland). The measurements for all airways were normalized to the internal perimeter (Pi) of the airway and averaged across all the sections for each subject. The Pi was measured by tracing along the luminal side of the epithelium. For the measurement of basement membrane thickness and collagen area, two additional sections were cut from each core (same as used for RNA isolation and other measurements) to stain with Masson’s trichrome (basement membrane) and Picrosirius red (collagen). To quantify the basement membrane thickness, a random series of line segments was placed over each image and the thickness of the basement membrane was measured at the points where any line segment crossed the basement membrane. All thickness measurements were made perpendicular to the epithelium. A minimum of 40 measurements were made for each airway. For the measurement of collagen content, Picrosirius red stained slides were visualized under polarized light where the Picrosirius red stain shows birefringence. The collagen appears red on a black background and the amount of collagen was quantified using color segmentation in Image Pro Plus®. All measurements were carried out in a blinded manner.

### Gene expression analysis

Expression of mRNA for the 334 candidate genes and 12 housekeeping genes was measured with the Nanostring® system using a custom codeset panel. The most stable housekeeping genes were selected by measuring 12 common housekeeping genes. By comparing the % Coefficient of variation (%CV) across the 12 housekeeping genes we were able to determine that the 5 most stable genes for data normalization were: RNA Polymerase R2A (POLR2A), TATA box binding protein (TBP), Ribosomal Protein L19 (RPL19), Guanine nucleotide-binding protein subunit beta-2-like 1 (GNB2L1), and β-Glucuronidase (GUSB). These 5 genes were also selected because they spanned a range of counts from low (average 198 counts for GUSB) to high (average 22,486 counts for RPL19). Data were normalized in accordance with Nanostring® guidelines. See Additional file 1: Table S2 in the online supplement for a complete list of genes in the panel.

### Candidate gene selection

We selected the candidate genes based on a priori hypotheses and grouped them based on their function and potential role in the pathogenesis of asthma and/or AHR. These are: ASM contraction and relaxation, structure and regulation of the cytoskeleton, epithelial barrier function, innate and adaptive immunity, fibrosis and remodeling and epigenetics. The rationale for the choice of groups of genes is provided below and the list of the genes by category is in Additional file 1: Table S2

1. **The contraction and relaxation of ASM and its regulation**; MYH11, MLCK, SM-22 and actin have been previously examined in the context of asthma [12]. Many of the genes in the contractile machinery group were taken from the Kyoto Encyclopedia of Genes and Genomes (KEGG) pathway for vascular smooth muscle contraction. Additionally, work by Sieck et al. [13] led to the selection of CD38 and other calcium handling proteins that have been shown to be expressed in cultured ASM cells. Genes within this pathway are involved in either contraction or relaxation of smooth muscle and so have the potential to play a role in AHR in asthma.
2. **The structure and regulation of the cytoskeleton**; genes within the cytoskeletal group were selected based on previous work by Gunst et al. [14]. Genes in this group have been shown to be important in transmitting ASM force to the external environment at adherens junctions, in maintaining the actin filament lattice or or regulating ASM stiffness independent of force generation [15].
3. **Epithelial barrier function**; a number of observations suggest that the airway epithelium is disrupted in asthma and that this may in part result from altered repair mechanisms [16]. Disrupted features include detachment of columnar ciliated cells, the presence of epithelial cell aggregates (Creola bodies) in sputum, decreased expression of epithelial cell-cell junction proteins (E-cadherin, ZO-1, protocadherin-1) and increased expression of epithelial repair markers (TGF-β, EGFR and CD44), mucins, and altered expression of repair-associated fucosylated glycoproteins [17]. A defective epithelial barrier may have important consequences in asthma as it is thought to lead to increased accessibility of allergens to immune and structural cells within the mucosal and submucosal spaces.
4. **Innate/Adaptive immunity**; innate immune receptors and related mediators have been implicated in the pathogenesis of asthma (e.g. IL-33, TSLP, and ST2) [18]. Furthermore, it has been demonstrated that antiviral immune responses are compromised in airway epithelial cells from asthmatics [19]. Intrinsic differences in innate immune responses in airway epithelial cells may therefore contribute to disease development and exacerbations in response to environmental exposures including allergens, viruses, and air pollution [20]. We therefore determined the expression patterns of all Toll-like receptors (TLRs), Nod-like receptors (NLRs), and Rig-like receptors (RLRs) and related mediators to provide a comprehensive screen of these candidate genes in asthmatic and non-asthmatic airway wall samples.
5. **Fibrosis and remodeling**; the role of the myocardin pathway in proliferation of ASM cells [21] led to the selection of genes in this pathway. In addition, we interrogated members of the Notch family as these genes are integral to airway development and differentiation [22] and may play important roles in asthma. ECM proteins are altered in the airways of asthmatics and as such, we included a number of genes that code for ECM components thought to be involved in the remodeling of asthmatic airways. In vivo work has shown a role for matrix metalloproteinases (MMPs) in the development of airway inflammation and hyperresponsiveness [23].
6. **Epigenetics**; as a first line of contact with the external environment, the airway epithelium is an attractive target for epigenetic research. Alterations in DNA methylation and histone modifications have been reported in the airway epithelium of asthmatic subjects [24], however many of the mediators involved have not been studied. We targeted the histone acetyltransferases KAT2A, CREBBP, and EP300 as they are responsible for acetylating lysine 18 on histone 3 which is up regulated in asthmatic epithelial cells [24]. We also focused on AURKA, PRMT5, SUV39H1, and HDAC10 which have been identified to be potentially involved in the pathogenesis of asthma based on preliminary data from an array analysis of epigenetic modifying enzymes (58). A number of genes chosen for this study were previously found to be differentially methylated in preliminary (PTK7, BCL3, DNMT3b, and PTPRO) [25] and final (CRIP1, STAT5A, FGFR1, S100A2, ITGA2, EGR4, EID1, and IGSF4C) analyses of DNA methylation in asthmatic airway epithelial cells [26].

Additional genes were added to the list given their reproducible association in asthma GWAS and observation that the SNP’s in these genes act as eQTLs.

### Data and statistical analyses

Final Nanostring results were filtered to keep only genes that had an average count of at least 30. Normalized mRNA expression values were compared between asthmatic and non-asthmatic subjects using a linear model with a negative binomial distribution controlling for age, sex, and inhaled corticosteroid use. Differential gene expression data are presented as volcano plots as well as in a summary table showing the top differentially regulated genes. The level of expression of each transcript is not completely independent since there was strong co-expression between the 344 genes. To account for this, we employed the Matrix Spectral Decomposition analysis of Nyholt and Li et al. [27, 28] to identify the effective number of independent genes. This lead to the multiple comparison correction shown in Table 2 that is based on an effective n of 31. Adjusted *p*-values will be listed as p.adj and genes with a nominal unadjusted *p* < 0.05 will be listed as p.unadj.

**Table 2:** Significant differentially expressed genes after *p*-value correction

Gene name	Symbol	Fold change	Counts (Asthma)	Counts (Non-Asthma)	Adjusted *p*-value (p.adj)
Integrin Beta 6	ITGB6	−1.51	367.4 ± 27.9	475.3 ± 46.4	0.002
Collagen Type 1 Alpha 1	COL1A1	1.92	1344.4 ± 222.0	735.8 ± 172.1	0.01
Collagen Type 3 Alpha 1	COL3A1	1.84	5324.3 ± 1517.9	3321.1 ± 321.3	0.03

In addition to the analysis of differential gene expression, we performed an analysis of differential co-expression in our data set using the analysis package CoXpress for R. Differential co-expression analysis identifies pairs of genes that are differentially co-expressed i.e. have opposite correlation patterns in cases vs. controls or show correlations in one condition only. Genes that were differentially co-expressed were entered into WebGestalt for pathway enrichment analysis and are presented in table form. Network analyst was used to understand the protein-protein interaction network [PMID: 25,950,236} of all nominally significant genes (p.unadj < 0.05). Data are plotted using GraphPad version 5.04 (La Jolla California USA).

## Results

### Airway characteristics

The total number of airways analyzed was 52 in asthmatic and 53 in non-asthmatic subjects; with an average of 4.3 airways per subject (*p* > 0.05, asthmatic vs. non-asthmatic). The average internal perimeter (Pi) of asthmatic subjects was 5.1 ± 1.5 mm (geometric mean 4.0 ± 1.7 mm) and in non-asthmatic subjects was 5.3 ± 1.5 mm (geometric mean 4.9 ± 1.3 mm) (p > 0.05 for both arithmetic and geometric mean). Airway wall area per unit length of Pi was significantly greater in asthmatics (0.22 ± 0.024 mm^2^/mm) than in non-asthmatics (0.13 ± 0.019 mm^2^/mm, *p* < 0.01). There was also an increase in the ASM area per unit Pi (0.018 ± 0.0024 mm^2^/mm vs. 0.011 ± 0.0015 mm^2^/mm, *p* < 0.05) and in basement membrane thickness (6.9 ± 0.81 mm vs. 3.9 ± 0.73 mm, *p* < 0.01) in asthmatics versus non-asthmatics. There was no significant difference in the area of epithelium or collagen per unit Pi between donor groups (*p* > 0.05). Side by side comparison of asthmatic and non-asthmatic airway can be seen in Fig. 2.

### Differential gene expression analysis

**Fig. 3:**
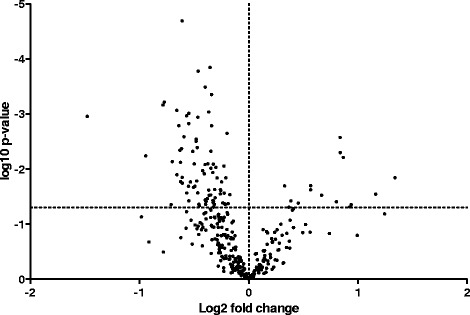
Volcano plot summarizing the results of the gene expression analysis. Dotted vertical line indicates fold difference of zero. Fold differences greater than zero (positive) indicate increased gene expressed in asthmatics compared to non-asthmatics. Dotted horizontal line indicates significance at nominal *p*-value of 0.05

**Fig. 4:**
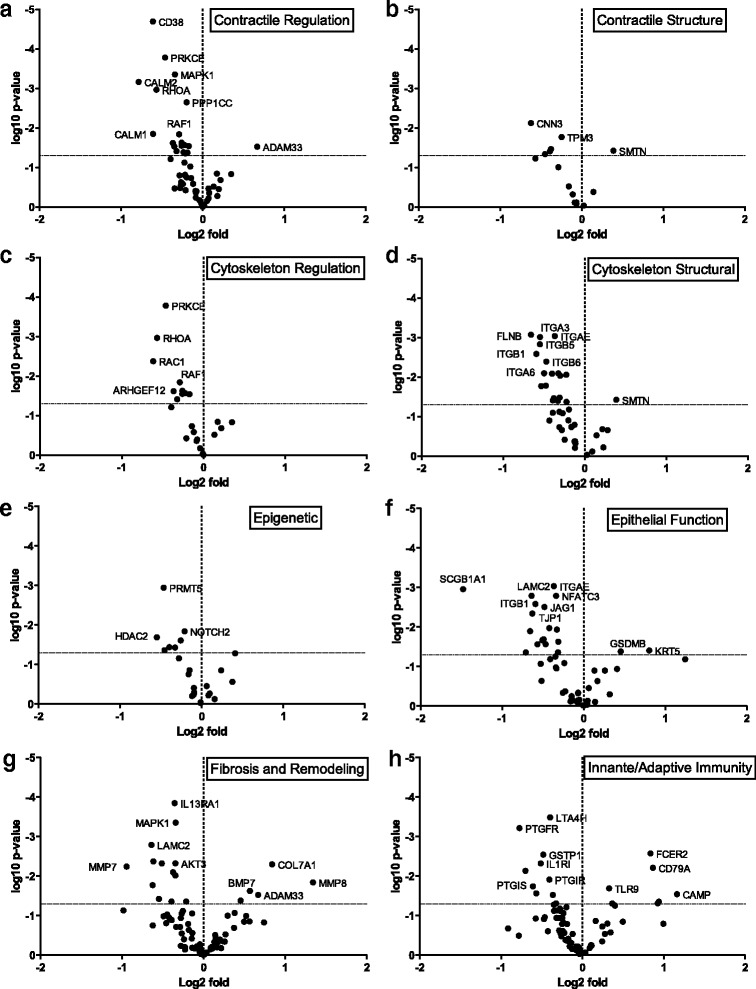
Volcano plots summarizing the results of gene expression for each hypothesis group. Genes involved in (**a**) Contractile regulation, (**b**) Structure of the contractile apparatus, (**c**) Cytoskeletal regulation, (**d**) Structure of the cytoskeleton, (**e**) Epigenetic control, (**f**) Epithelial function, (**g**) Fibrosis and remodeling, (**h**) Innate and adaptive immunity. Some genes fall into more than one category and so are plotted in applicable categories. Dotted vertical line indicates fold differences of zero, dotted horizontal lines indicates significance at nominal *p*-value of 0.05

Gene expression changes in all genes are summarized in Fig. 3 with the candidate gene hypothesis categories plotted in Fig. 4. In total there were 51 genes differentially expressed based on a threshold *p*-value of *p* < 0.05 and three genes that were significant after p-value correction (Table 2, p.adj). In brief, there were three genes whose significance reached the adjusted p-value cutoff, Collagen Type 1 Alpha 1 (*COL1A1*), *COL3A1*, and integrin beta 6 (*ITGB6*). The gene for *COL1A1* was the most significantly up-regulated gene (1.83-fold increase, p.adj = 0.01) and integrin beta 6 (*ITGB6*) was the most significantly down-regulated gene (1.29-fold decrease, p.adj = 0.002) (Additional file 1: Table S2). COL1A1 expression was positively associated with the amount of collagen in the airway in asthmatics and non-asthmatics (Collagen/Pi, R^2^ = 0.2221 and 0.2182 for asthma and non-asthma respectively, p < 0.01) and the thickness of the basement membrane in both groups combined (R^2^ = 0.1313, *p* = 0.01). COL3A1 expression was positively associated with ASM/Pi (R^2^ = 0.2089, *p* = 0.02) and Collagen/Pi (R^2^ = 0.2083, *p* = 0.03) in asthma. ITGB6 expression was negatively associated with basement membrane thickness in asthma (R^2^ = 0.1801, *p* = 0.04) and negatively associated with Epithelial area/Pi in both groups combined (R^2^ = 0.1158, p = 0.02). There was an association between ITGB6 expression and Collagen/Pi in asthma that did not quite reach significance. (R^2^ = 0.144, *p* = 0.06). In each hypothesis group there were a number of differentially expressed genes that did not reach significance after *p*-value adjustment, these included: contractile apparatus structure – Smoothelin (1.40 fold increase, p.unadj = 0.01); regulation of contraction – CD38 (1.66 fold decrease, p.unadj =0.003); cytoskeletal structure – ITGB6 (see above); cytoskeletal regulation – RAC1 (1.60 fold decrease, p.unadj =0.002); epigenetic regulation – PRMT5 (1.52 fold decrease, p.unadj =0.03); epithelial function – LAMC2 (1.59 fold decrease, p.unadj =0.006); fibrosis and remodeling – COL1A1 (see above); innate and adaptive immunity – PTGFR (2.18 fold decrease, p.unadj =0.004). The counts, *p*-values, and adjusted *p*-values for all significant genes can be seen in Additional file 1: Table S2. Of the 12 genes identified in GWAS or linkage analysis, only *ADAM33* (1.56-fold increase, p.unadj =0.0057) was up-regulated. There were no pathways significantly enriched in the differentially up or down-regulated genes.

**Fig. 5:**
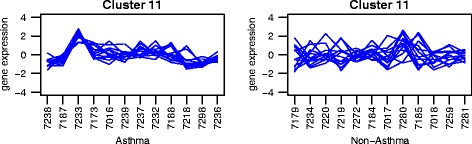
Example of co-expression plots. Each line represents one gene in the cluster. Subjects are indicated along the x-axis, log expression values on the y-axis. *P*-values are for the significance of the co-expression in each group

Using Network Analyst, we identified a minimum protein-protein interaction network and key nodes from our nominally differentially expressed genes (Fig. 5). Green nodes indicate down-regulated genes, red nodes indicate up-regulated nodes (both relative to non-asthmatics), and grey nodes indicate first order interactions. This network highlights a number of key nodes in our data set including: Mitogen-Activated Protein Kinase 1 (MAPK1, degree = 24), c-FOS (degree = 22, and Calmodulin 3 (CALM3, degree = 21). Using this network, we were able to identify pathways significantly associated with up and down-regulated nodes (Table 3). These included key pathways in collagen degradation and remodeling and alterations to cell-cell communication.

**Table 3:** Pathways significantly associated with protein-protein interaction nodes

Pathway name	Hits/Total	FDR
Up-Regulated Pathways		
Degradation of Collagen	4/61	0.003
Extracellular Matrix Organization	5/157	0.003
Degradation of Extracellular Matrix	4/77	0.004
Assembly of Collagen Fibrills and Other Multimeric Structures	3/54	0.04
Collagen Biosynthesis and Modifying Enzymes	3/62	0.04
Down-regulated pathways		
Integrin Cell Surface Interactions	6/85	0.0007
Signal Transduction	17/1690	0.004
Cell-Cell Communication	6/143	0.004
Platelet Activation, Signaling, and Aggregation	7/220	0.004
TGF-beta Receptor Signaling in EMT	3/17	0.009
Hemostatis	9/511	0.01
Signaling by TGF-beta Receptor Complex	4/70	0.02
Sema4D Induced Cell Migration and Growth-Cone Collapse	3/29	0.03
Sema4D in Semaphorin Signaling	3/34	0.04
Fc-gamma Receptor Dependent Phagocytosis	4/86	0.04

### Differential co-expression analysis

We identified groups of genes that were differentially co-expressed between asthmatics and non-asthmatics. In this analysis, genes are clustered together based on how their expression values correlate with each other. The analysis was performed twice with the comparison group being the non-asthmatics or asthmatics in the different analyses. Clusters of genes that are significantly co-expressed in one condition and not in the other are said to be differentially co-expressed. Figure 5 shows an example; expression of genes in cluster 11 changes from subject to subject in asthmatics (left) and non-asthmatics (right). Each line represents one gene in the group. Genes in cluster 11 follow a similar pattern of expression in asthmatics (*p* < 0.001) but not in non-asthmatics (*p* = 0.32). The rest of these figures can be seen in Additional file 1: Fig. S3 and S4.

**Fig. 6:**
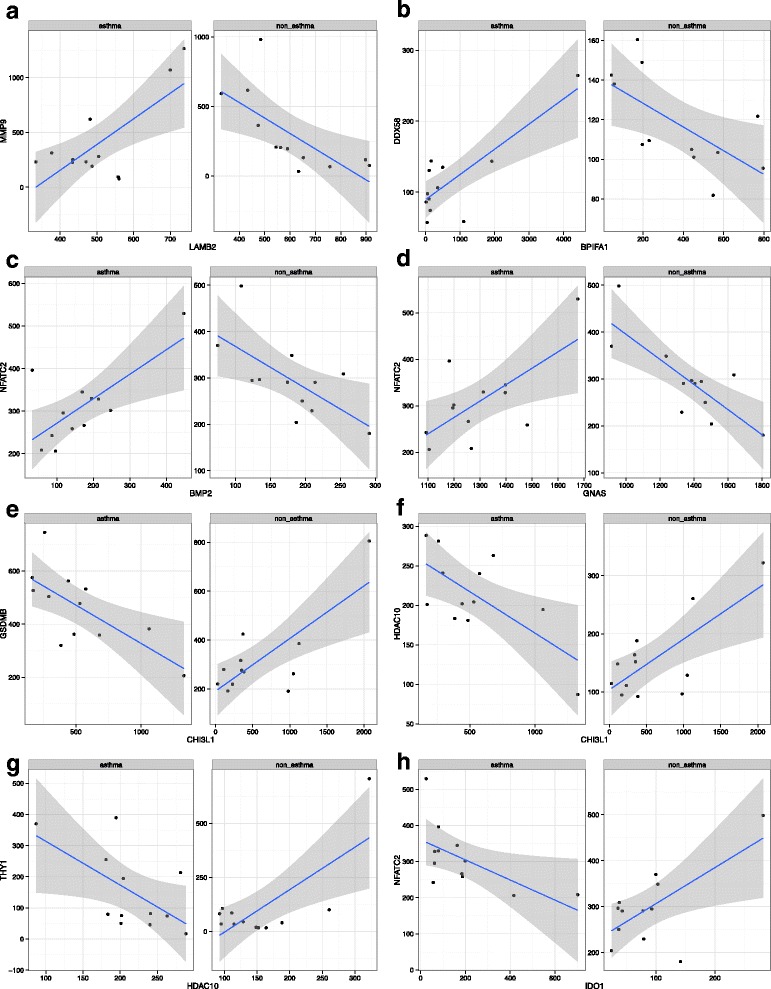
Individual correlation plots for the pairs of differentially co-expressed genes in asthmatics and non-asthmatics. **a** LAMB2 vs. MMP9, (**b**) BPIFA1 vs. DDX58, (**c**) BMP2 vs. NFACT2, (**d**) GNAS vs. NFACT2, (**e**) CHI3L1 vs. GSDMB, (F) CHI3L1 vs. HDAC10, (G) HDAC10 vs. THY1, (F) IDO1 vs. NFATC2. Each point represents a sample and the values on the axes are gene counts. Lines of best fit are plotted with the 95% confidence interval (grey shaded area) for each correlation. Each of the correlations is significant (*p* < 0.05) and the direction indicates a positive or negative correlation

In non-asthmatics, there were 3 groups of genes that were differentially co-expressed compared to asthmatics. These clusters (10, 35, and 53) had an average co-expression correlation coefficient of 0.772 (*p* < 0.0001) in non-asthmatics and 0.223 (*p* > 0.05) in asthmatics. The genes in these groupings are summarized in Additional file 1: Table S3. We performed pathways analysis on each group of genes with Webgestalt. These results are shown in Table 4. There were no pathways that were significantly enriched for genes differentially co-expressed in non-asthmatics. Further analysis identified specific gene pairs from cluster 10 that were both significantly positively correlated in non-asthmatics and significantly negatively correlated in asthmatics (Table 5). These included: chitinase 3-like 1(*CHI3L1*) and *GSDMB* (*R* = 0.760 and *R* = −0.707); *CHI3L1* and histone deacetylase 10 (*HDAC10*) (*R* = 0.731 and *R* = −0.676); *HDAC10* and thymocyte antigen 1 (*THY1* or *CD90*) (*R* = 0.727 and *R* = −0.596); and indoleamine 2,3-dioxygenase 1 (*IDO1*) and nuclear factor of activated T-cells, cytoplasmic 2 (*NFATC2*) (*R* = 0.659 and *R* = −0.604), in non-asthmatics and asthmatics respectively. All showed significant but opposite direction of correlation (*p* < 0.05 Fig. 6).

**Table 4:** Pathways enriched in differentially co-expressed genes. *p*-value comes from using list of 334 genes as background

Coexpressed in Non-asthmatics				
Cluster #	# Genes in Cluster	# Genes From Cluster in Pathway	Biological Process	*p*-value
10	25	3	Regulation of Cell-Cell Adhesion Involved in Gastrulation	2.0 × 10^−1^
		13	Regulation of Multicellular Organismal Development	2.0 × 10^−1^
		8	Regulation of Cell Adhesion	2.0 × 10^−1^
53	6	4	Cell Migration	4.0 × 10^−1^
		4	Locomotion	5.7 × 10^−1^
		4	Localization of Cell	4.0 × 10^−1^
Coexpressed in Asthmatics				
16	6	3	**Cytoplasmic Pattern Recognition Receptor Signaling Pathway in Response to Virus**	**3.0 × 10** ^**−4**^
		3	**Positive Regulation of Type 1 Interferon Production**	**3.9 × 10** ^**−3**^
		3	**Negative Regulation of Type 1 Interferon Production**	**2.0 × 10** ^**−3**^
20	6	4	Activation of MAPK Activity	6.9 × 10^−2^
		4	Peptidyl-Tyrosine Phosphorylation	7.9 × 10^−2^
		3	Eicosanoind Biosynthetic Pathway	7.9 × 10^−2^
11	9	3	Odontogenesis	4.2 × 10^−1^
17	7	4	Response to Bacterium	3.9 × 10^−1^

Bolded pathways highlight those that reached statistical significance in the co-expression data set

**Table 5:** Pairs of differentially co-expressed genes

Gene 1	Gene 2	Asthma R	*p*-value	Non-Asthma R	*p*-value
LAMB2	MMP9	0.727	0.007	−0.686	0.013
BPIFA1	DDX58	0.814	0.001	−0.646	0.023
BMP2	NFATC2	0.707	0.010	−0.660	0.020
GNAS	NFATC2	0.665	0.018	−0.798	0.002
CHI3L1	GSDMB	−0.707	0.010	0.760	0.004
CHI3L1	HDAC10	−0.676	0.016	0.731	0.007
HDAC10	THY1	−0.596	0.041	0.727	0.007
IDO1	NFATC2	−0.604	0.037	0.659	0.0198

In asthmatic samples there were 6 clusters of genes that were found to be differentially co-expressed. These clusters had an average correlation coefficient of 0.728 (p < 0.0001) in asthmatics and 0.169 (p > 0.05) in non-asthmatics. The genes in these 6 clusters are summarized in Additional file 1: Table S3. Each of the clusters was also analyzed with Webgestalt (Table 4). In brief, asthmatic co-expressed genes were significantly enriched in pathways for cytoplasmic virus pattern recognition signaling (*p* = 3.0 × 10^−4^), positive, and negative regulation of type 1 interferon production (*p* = 3.9 × 10^−3^, and *p* = 2.0 × 10^−3^ respectively). Within the cluster of genes that were differentially co-expressed, there were 4 pairs of genes whose expression were positively and significantly correlated in asthmatics but negatively and significantly correlated in non-asthmatics (Table 5). In brief: laminin B2 (*LAMB2*) and matrix metallopeptidase 9 (*MMP9*) (R = 0.727 and *R* = −0.686); BPI fold containing family A (BPFIA1) and DEAD box polypeptide 58 (*DDX58* or *RIG-1*, retinoic acid inducible gene 1 protein) (*R* = 0.814 and *R* = −0.646); bone morphogenetic protein 2 (*BMP2*) and *NFATC2* (*R* = 0.707 and *R* = −0.660); and G-protein alpha stimulating (*GNAS*) and *NFATC2* (*R* = 0.665 and *R* = −0.798), in asthmatics and non-asthmatic respectively. All were significantly correlated but in opposite directions (p < 0.05, Fig. 6).

All eight pairs of differentially co-expressed genes (Table 5) were entered into the ENCODE ChIP-SEQ significance tool. All but one of the genes (*IDO1*) from the pairs was regulated by the transcriptional repressor CCCTC-binding factor or CTCF. Since only 47% of the candidate genes have a CTCF binding site this represents a significant enrichment (*p* = 0.0035, Chi-squared test).

## Discussion

In this study the Nanostring® platform was used to quantify the airway expression of candidate genes hypothesized to be important in the pathophysiology of asthma. mRNA was obtained from the airways of asthmatic (mainly fatal asthmatics) and non-asthmatic donor lungs and measures of airway remodeling were made on the sampled airways. Candidate genes were grouped into the following categories: ASM contraction, the cytoskeleton, epithelial barrier function, innate/adaptive immunity, fibrosis/remodeling, and epigenetics. 51 genes (15%) were nominally differentially expressed (p.unadj <0.05) in asthmatic airway tissue and included many genes important in cell-cell and cell-matrix interactions (COL1A1, COL3A1, ITGB6, LAMC2, RAC1). Of the 51 genes differentially expressed based on a nominal *p*-value, only 3 were significant following multiple comparison correction (ITGB6, COL1A1, COL3A1).

Cell-cell junctions are altered in the airway epithelium of asthmatics [29] and this may result in greater permeability of the epithelial layer and ultimately hypersensitivity of the ASM to agonist challenge [30]. The most significant differentially expressed gene in the data set was ITGB6. ITGB6 rapidly accumulates following injury to the epithelial layer and is considered to be important for normal wound healing [31]. In the mouse loss of ITGB6 causes an increase in the number of B-cells and T-cells around the airways, an increase in IL-4 production, and airway hyperresponsivenss in naïve mice without allergen challenge [31]. Additionally, influenza virus has been shown to interact with ITGB6 to cause epithelial cell death and collagen deposition in a TGF-ß dependent manner [32]. Another gene with lower abundance in asthmatics that did not meet the adjusted *p*-value cut off was RAC1. RAC1 has been shown to be important in the formation of tight junctions in an EGFR dependent manner [33], specifically by regulating tight junction protein 1 (or zona occluden 1) [34] which was also lower in abundance in the asthmatic samples. Changes in these genes could also affect epithelial-mesenchymal transition (EMT) [35], cytoskeletal stability, actin filament assembly/disassembly, cell stiffness and/or cell migration [36]. In addition, a protein-protein interaction network highlighted that down-regulated genes were enriched in pathways for cell-cell communication and integrin cell surface interactions.

The most significantly up-regulated gene in asthmatic samples was *COL1A1* which codes for the alpha chain in type 1 collagen. Collagen 1 is the major type of collagen in basement membrane and a major protein found in remodeled airways. Collagen 1 is important in airway remodeling [37], in particular thickening of the subepithelial space which is associated with worsening of asthma symptoms [38]. Collagen type 3 was also significantly elevated in asthmatic subjects and is also significantly elevated in the basement membrane of asthmatic subjects [39]. Type 1 and 3 collagen have both been associated with worsening lung function in a horse model of asthma [40]. Beyond the ability of collagens to affect distensibility of the airways, collagen 1 has been shown to stimulate ASM to produce MMP1 [41] and proliferate, in conjunction with FAK [42]. Collagen 1 and 3 expression has also been shown to be unaffected by corticosteroid usage in severe asthmatics [43] and collagen 1 and 3 contribute to the loss of the anti-mitotic effect of corticosteroids [44]. Enrichment of pathways involved in collagen remodeling was seen in our network analysis and highlights the importance of understanding fibrosis as it relates to fiber production, degradation, and organization and how this impacts normal cell function.

*ADAM33* has been described in candidate gene studies to be associated with asthma [6] and was significantly elevated in asthmatic airways. *ADAM33* has been implicated in smooth muscle development, cell-cell connections, and cell proliferation and differentiation [45]. Over-expression of *ADAM33* in asthmatic airways has been described [46] and may be an important determinant of disease progression. There is evidence that ADAM33 can stimulate angiogenesis ex vivo and in vivo and by this mechanisms may contribute to airway remodeling [47]. Furthermore, ADAM33 family member TACE/ADAM17 can mediate release of TNF-α and fracktalkine (or CX3CL1) from the cell membrane [48, 49] and ADAM9 may mediate the release of growth factor HB-EGF [50]. If ADAM33 has a similar capacity for cytokine or growth factor cleavage this could make it a major contributor to airway remodeling in asthma.

Other genes in the 5 most differentially up or down regulated genes that were nominaly significant include: Cyclic ADP Ribose (CD38), Interleukin 13 Receptor Alpha 1 (IL13RA1), Prostaglandin F Receptor (PTGFR), Heat Shock Protein Beta 1 (HSPB1), and Interferon Induced with Helicase C Domain 1 (IFIH1). CD38 is a protein that generates the second messenger cADPR to cause calcium release. Recent work has explored the role of CD38 in asthma and has suggested that increased CD38 expression causes hypercontractility in ASM cells from asthmatics [51] although in our samples we saw no CD38 staining in the ASM layer (Additional file 1: Fig. S1 and S2). Additionally, CD38 deficient mice have reduced AHR following ovalbumin challenge [52]. Decreased IL13RA1 expression is surprising in the context of asthma but this could be due to a compensatory response to continued eosinophilia and IL13/IL4 exposure in the lung [53]. Prostaglandin’s can be both pro and anti inflammatory but there is little research on the role of prostaglandin F in the context of asthma. HSPB1 (or heat shock protein 27) is a chaperone protein that has been implicated in cellular differentiation, apoptosis, and smooth muscle contraction [54]. Up-regulation of the gene could contribute to ASM hypercontractility in asthma but further work investigating the phosphorylation state and activity of HSP27 in asthma is needed to answer this question. IFIH1, also known as MDA5, is a DEAD box double stranded (ds) RNA helicases that can detect intracellular viral dsRNA and lead to the production of interferons [55]. MDA5 and TLR3 signaling have been shown to be deficient in bronchial epithelial cells from asthmatic subjects [56] and this could be responsible for the defective epithelial release of interferon I and III in response to rhino virus infection [57, 58].

Co-expression of genes does not imply interaction between their proteins but instead may suggest similarities in their regulation by transcription factors or epigenetic mechanisms [59]. Co-expression analyses can reveal changes in the regulation of gene expression [60] and have been used to identify epigenetic changes that affect gene co-expression in cancer [61]. In our study, genes that were differentially co-expressed between asthmatics and non-asthmatics were significantly enriched for pathways involved in virus recognition and regulation of interferon production (Table 4). The genes enriched in these pathways were from cluster 16 and were RIG-I (*DDX58*), RIG-1-like receptor 3 (*DHX58*), and interferon induced with helicase C domain 1 (*IFIH1*). This finding suggests that a central molecular mechanism may regulate diverse antiviral immune molecules in response to viral infections that may trigger asthma exacerbations and/or pathogenesis [62]. IFIH1, as discussed earlier, was also differentially expressed.

One of the most intriguing results was the identification of a single transcriptional repressor, CTCF, that controls the expression of all but one of the differentially co-expressed pairs of genes. CTCF influences gene expression through chromatin modifications [63] resulting in insulation of the target regions [64]. CTCF is an architectural protein that mediates inter- and intra-chromosomal interactions at distant genomic sites, and regulates three-dimensional genome architecture [63]. There are examples of CTCF silencing one gene while activating another [63]. Specific to asthma, differential expression at the *ZPBP2*/*GSDMB*/*ORMDL3* locus was identified resulting from allele-specific chromatin remodeling mediated by CTCF [65]. A SNP in *ZPBP2* created a CTCF binding site resulting in increased expression of ZPBP2 but diminished expression of *GSDMB* and *ORMDL3* [65]. Additionally, CTCF is highly sensitive to DNA methylation at CTCF binding sites [63]; changes to the methylome can have direct effects on the regulation of CTCF target genes. CTCF could play a crucial role in controlling the many gene expression changes observed in the airways of asthmatics and is worthy of more intense research that is beyond the scope of this paper.

There are several limitations of this study. Firstly, the use of whole airway RNA rather than RNA from specific cell types precludes us from conclusively identifying the site of gene expression. Secondly, the majority of the asthmatic patients were fatal asthmatics and experienced hypoxia and treatment with steroids during their fatal attack which can affect mRNA expression in tissues taken for research purposes. We addressed this by controlling for steroid use in our analysis of differential gene expression. Additionally, the use of more severe asthmatics may mean that these results are not generalizable to asthmatics as a whole. But considering severe asthmatic populations have the most hospital visits and are most at risk for exacerbations, we believe our results provide significant insight into the genes that are altered in fatal disease. Finally, the relatively small sample size limits our ability to detect differences in gene expression less than ~1.5 fold on average, although this also means that the changes we see are likely to be real. Procurement of donor lungs is time consuming and costly so increasing the number of patients for this study was not feasible, however future studies in asthmatic biopsies or cell culture experiments could confirm these results with the ability for much larger sample size. A limited number of donors with non-fatal asthma (*n* = 4) means we were unable to test for differences between these two groups of donors (fatal vs. non-fatal).

## Conclusion

This study identifies changes in the expression and co-expression of genes thought to be important in asthma and AHR. Specifically, we identified altered abundance of genes involved in cell-cell and cell-matrix connections as well as those involved in the immune response and cell homeostasis. We also identified changes in the co-expression of genes involved in virus recognition and interferon production. The transcription factor CTCF could be an important regulator of the asthmatic phenotype and warrants further investigation. Future work should focus on elucidating the potential mechanisms behind altered CTCF binding as it relates to asthma pathophysiology.

## Additional file

Additional file 1: Supplementary Methods – Methods describing selection of house keeping genes and immunohistochemical staining procedure. Supplementary Tables – Tables containing clinical demographics for subjects, average counts, fold change, and *p*-value for all genes studied, and all differentially co-expressed genes. Supplementary Figures and Legends – Figures showing sample immunohistochemical staining for proteins of significantly altered genes, co-expression plots. (DOCX 35 kb)

## Abbreviations

%CV: Percent coefficient of variation
ADAM17: ADAM metallopeptidase domain 17
ADAM33: ADAM metallopeptidase domain 33
ADAM9: ADAM metallopeptidase domain 9
AHR: Airway hyperresponsiveness
ASM: Airway smooth muscle
AURKA: Aurora kinase A
BCL3: B-Cell CLL/lymphoma 3
BMP2: Bone morphogenetic protein 2
BPIFA1: BPI fold containing family A member 1
cADPR: Cyclic ADP Ribose
CD38: Cluster of differentiation 38
CD44: Cluster of differentiation 44
CHI3L1: Chitinase 3-like
COL1A1: Collagen Type 1 Alpha 1
COL3A1: Collagen Type 3 Alpha 1
CREBBP: CREB binding protein
CRIP1: Cysteine rich protein 1
CTCF: CCCTC-binding factor
CX3CL1: Fracktalkine
DDX58: DExD/H-box helicase 58
DNA: Deoxyribonuleic acid
DNMT3b: DNA methyltransferase 3 Beta
ECM: Extracellular matrix
EGFR: Epidermal growth factor receptor
EGR4: Early growth response 4
EID1: EP300 interacting inhibitor of differentiation 1
EMT: Epithelial-mesenchymal transition
EP300: E1A binding protein P300
eQTL: Expression quantitative trait loci
FAK: Focal adhesion kinase
FGFR1: Fibroblast growth factor receptor 1
GNAS: Adenylate cyclase-stimulating G alpha protein
GNB2L1: Guanine nucleotide-binding protein subunit Beta-2-Like 1
GSDMB: Gasdermin B
GUSB: β-Glucuronidase
GWAS: Genome wide association study
H&E: Hemoatoxylin and eosin
HB-EGF: Heparin-binding EGF-like growth factor
HDAC10: Histone deacetylase 10
HSPB1: Heat shock protein 27
IDO1: Indoleamine 2,3-dioxygenase 1
IFIH1: Interferon induced with helicase C domain 1
IGSF4C: Immunoglobulin superfamily member 4C
IIAM: International Institute of the Advancement of Medicine
IL-13: Interleukin-13
IL33: Interleukin-33
IL4: Interleukin-4
ITGA2: Integrin subunit Alpha 2
ITGB6: Integrin subunit Beta 6
KAT2A: Lysine acetyltransferase 2A
KEGG: Kyoto encyclopedia of genes and genomes
LAMB2: Laminin subunit Beta 2
LAMC2: Laminin subunit Gamma 2
MDA5: Melanoma differentiation-associated protein 5
MLCK: Myosin light chain kinase
MMP1: Matrix metallopeptidase 1
MMP9: Matrix metallopeptidase 9
MYH11: Myosin heavy chain 11
NFATC2: Nuclear factor of activated T-cells, cytoplasmic 2
NLR: Nod-like receptor
ORMDL3: ORMDL sphingolipid biosynthesis regulator 3
Pi: Internal perimeter of the airway
POLR2A: RNA polymerase R2A
PRMT5: Protein arginine methyltransferase 5
PTGFR: Prostaglandin F receptor
PTK7: Protein tyrosine kinase 7
PTPRO: Protein tyrosine phosphatase, receptor type O
RAC1: Ras-related C3 botulinum toxin substrate 1
RIG-1: Retinoic acid inducible gene 1
RLR: Rig-like receptor
RNA: Ribonucleic acid
RPL19: Ribosomal protein L19
S100A2: S100 calcium binding protein A2
SEM: Standard error of the mean
SM-22: Smooth muscle protein 22-alpha/transgelin
SNP: Single nucleotide polymorphism
ST-2: Suppression of tumorigenicity 2
STAT5A: Signal transducer and activator of transcription 5A
SUV39H1: Suppressor of variegation 3–9 homolog 1
TACE: Tumor necrosis factor-α-converting enzyme
TBP: TATA box binding protein
TGF-β: Transforming growth factor Beta 1
THY1: Thymocyte antigen 1
TLR: Toll-like receptors
TNF- α: Tumor necoris factor alpha
TSLP: Thymic stromal lymphopoietin
ZO-1: Zona occludens-1
ZPBP2: Zona pellucida binding protein 2
