# Supplementary material for: Gene expression analysis in asthma using a targeted multiplex array
Source: BMC Pulm Med. 2017 Dec 11;17:189. doi: 10.1186/s12890-017-0545-9 (PMC5725935; doi:10.1186/s12890-017-0545-9)
Supplement: Additional file 1: — Supplementary Methods – Methods describing selection of house keeping genes and immunohistochemical staining procedure. Supplementary Tables – Tables containing clinical demographics for subjects, average counts, fold change, and p-value for all genes studied, and all differentially co-expressed genes. Supplementary Figures and Legends – Figures showing sample immunohistochemical staining for proteins of significantly altered genes, co-expression plots. (DOCX 35 kb) [file 12890_2017_545_MOESM1_ESM.docx]

**Supplementary Text, Figures, and Tables**

**Determination of most stable housekeeping genes**

Housekeeping genes were selected by conducting a separate study using 3 asthmatic and 3 non-asthmatic airway sections in technical duplicates. Total RNA isolated from the whole tissue (airway, parenchyma and vessels) was sent to Nanostring® for analysis. Using the reference panel from Nanostring®, the expression of 18 commonly used housekeeping genes was measured and the percent coefficient of variation (%CV) compared across the 6 subjects. The top 4 genes that had the lowest %CV were selected for the final gene expression panel. These were: RNA Polymerase R2A (POLR2A – 36.3% CV), TATA box binding protein (TBP – 40.1% CV), Ribosomal Protein L19 (RPL19 – 53.9% CV), and β-Glucuronidase (GUSB, 57.6% CV). These 4 genes were also selected because they spanned a range of counts from low (average 198 counts for GUSB) to high (average 22,486 counts for RPL19). These 4 housekeeping genes were added to another 8 for a total of 12 housekeeping genes in the final panel. This was done so that we could select the best housekeeping genes for the study. The 4 originally selected best housekeeping genes were in the top 5 for lowest %CV values and an additional housekeeping gene not originally in the 18 from the separate study was added since it fell within the top 5 housekeeping genes. The 5 housekeeping genes used in this study and their %CV are: POLR2A (14.6%), RPL19 (16.2%), GUSB (17.1%), GNB2L1 (Guanine nucleotide-binding protein subunit beta-2-like 1, 19.3%), and TBP (20.0%). Again these 5 housekeeping genes spanned a range of counts from low to high. Genes in the codeset were normalized to the geometric mean of these 5 housekeeping genes.

**Immunohistochemical Staining Procedure**

Protein expression and localization of Filamin B (FLNB), CD38 and Smoothelin (SMTN) was verified by immunohistochemistry (IHC) on formalin fixed paraffin embedded (FFPE) sections from each of the 24 subjects used for RNA analyses. The antibodies for FLNB (ab84905), CD38 (ab93941) and SMTN (ab8969) were ordered from Abcam® (Cambridge UK). Sections of 4μm thickness were cut from the lung cores. FFPE sections were deparaffinized in CitriSolv (Fisher Scientific) and rehydrated using a graded series of alcohol. Sections were then autoclaved (Bench Top Tuttnauer 2340M autoclave) on a 22-minute cycle in Citra buffer, pH6, for antigen retrieval and were then left to cool for 20 minutes. All the IHC staining was performed on a Dako Autostainer Universal Staining System and all incubations throughout the staining run were carried out at room temperature. After cooling, the slides were loaded onto the Autostainer and incubated in Background Sniper (BS966: Biocare Medical; Concord, CA, USA) for 15 minutes to help reduce non-specific binding. The sections were incubated with the primary antibody at 1/200 dilution for 45 minutes. Detection was performed using the MACH3 Universal AP staining kit (Biocare Medical). Slides were covered with MACH3 Universal AP Polymer (M4U536) for 10 minutes each. Finally, the sections were incubated with Vulcan Fast Red as chromagen (Biocare, FR805) for 12 minutes and then counterstained with diluted Gill’s III Hematoxylin (SurgiPath) for 2 minutes. As negative control, normal mouse serum was used at the same concentration as the primary antibody. Positive controls were as follows: CD38 and FLNB – lung carcinoma, SMTN – vascular smooth muscle. The degree of staining was quantified for the whole airway, ASM bundles and epithelial layer.

**Quantification of Immunohistochemical Staining**

IHC staining was quantified using the positive pixel count algorithm that is part of the Aperio Image Scope® software. The area of interest for each of the analyzed layers (whole airway, ASM, epithelium) were traced to confine the algorithm to the correct tissue compartment. The algorithm variables, which were set to minimize non-specific identification of staining, were determined separately for each protein of interest. This was done by iteratively applying the algorithm to both the positive and negative controls and maximizing the selection of the positive area while minimizing the non-specific signal on the negative slide. The hue value, width and saturation levels where repeatedly adjusted until the ratio of the signal in the positive control and negative control was maximized. Hue values usually centered around a value of 0.98 on a color wheel where 0 is red, 0.33 is green and 0.66 is blue. Hue widths and saturation levels where set for each individual stain and were dependent on the shade of pink on the slide with pale pink needing lower saturation values or a wider range of hues and vibrant pinks needing a higher saturation value or narrower range of hues. For each tissue compartment analyzed, the percent positivity was recorded. The percent positivity is the percentage of the traced area that is positive for the color of interest (in this case pink). One section per subject was stained and analyzed this way. All measurements were carried out in a blinded manner.

Supplementary Table S1 – Subject Clinical Details

| **Condition** | **Sex** | **Age** | **Weight (kg)** | **Height (cm)** | **Ethnicity** | **Cause of Death** | **Medical History** | **Drug History** |
| --- | --- | --- | --- | --- | --- | --- | --- | --- |
| Asthma | F | 8 | 30.5 | 117 | Hispanic | Asthma attack | Asthma diagnosed age 3. RSV age 2 | Albuterol, Singular, |
| Asthma | M | 11 | 65.3 | 152.5 | Caucasian | Head Trauma | Asthma diagnosed age 6 | N/A |
| Asthma | F | 21 | 55 | 170 | Caucasian | Drug Overdose (Tyleonl) | Asthma; Cervical Cancer free 1 year; Cigarettes 2PPD for 6 years | Albuterol, Advair |
| Asthma | F | 15 | 56 | 162.5 | Caucasian | Anoxia, probable asthma attack | Environmental allergies and asthma; smoked cigarettes <1PPD for 1 year; Marijuana 2 times a week | Singular, Advair, Flovent, Albuterol, Prednisone |
| Asthma | M | 11 | 69 | 170 | Caucasian | Anoxia, probable asthma attack | Asthma diagnosed age 2 | Albuterol |
| Asthma | F | 26 | 91.6 | 168 | Caucasian | Anoxia, probable asthma attack | Asthma diagnosed in childhood; seizure in 5th grade; frequent UTI; cigarettes 1 pack/week since age 16; hard liquor, heavy drinker | Albuterol, Advair |
| Asthma | M | 10 | 43 | 157.5 | Caucasian | Asthma attack | Asthma diagnosed age 4 | Advair, Albuterol |
| Asthma | M | 25 | 103 | 185 | Hispanic | Anoxia, suicide | Asthma diagnosed age 11; cigarettes 2 PPD for 12 years; Marijuana 6x/day for 12 years | Albuterol, Advair |
| Asthma | F | 15 | 67 | 177.8 | Caucasian | Asthma attack | Asthma | Prednisone, Albuterol |
| Asthma | M | 36 | 91 | 173 | Caucasian | Head Trauma | Asthma; Chewing Tobacco daily; Alcohol occasionally in large amounts | Inhaler (unknown type) |
| Asthma | M | 23 | 52.6 | 152.4 | Hispanic | Asthma attack | Asthma diagnosed at birth; Seizures 2-3x age 1 & 2 | Inhaler (unknown type) |
| Asthma | M | 20 | 81.2 | 172.7 | Caucasian | Asthma | N/A | N/A |
|  |  |  |  |  |  |  |  |  |
| Non-asthmatic | M | 20 | 86 | 185 | Caucasian | Head Trauma, MVA | Cigarettes 2/week; Vodka half bottle/week; Marijuana weekly | None |
| Non-asthmatic | F | 4 | 17.5 | 104 | Hispanic | Head Trauma | None | None |
| Non-asthmatic | M | 22 | 112 | 193 | Caucasian | Head Trauma | Occasional marijuana | None |
| Non-asthmatic | F | 63 | 77 | 159 | Caucasian | Gastrointestinal Bleed | Hypertension for 2 years | Unknown Antihypertensive |
| Non-asthmatic | M | 14 | 50 | 165 | Caucasian | Head Trauma, MVA | None | None |
| Non-asthmatic | F | 19 | 87 | 165 | Caucasian | Head Trauma, MVA | Beer/Hard Liquor 1-2x/month for 2 years | None |
| Non-asthmatic | F | 20 | 72.2 | 165 | Caucasian | Head Trauma | Cigarettes <1PPD for 2 years, Marijuana smoked unknown frequency, Pain medication and inhalents | None |
| Non-asthmatic | M | 24 | 81.8 | 175 | Hispanic | Head Trauma | None | None |
| Non-asthmatic | M | 12 | 48.1 | 160 | Caucasian | Head Trauma, MVA | None | None |
| Non-asthmatic | F | 42 | 90 | 178 | Caucasian | Head Trauma, MVA | Hypertension diagnosed age 41 | None |
| Non-asthmatic | F | 47 | 82 | 167.5 | Caucasian | CVA | N/A | None |
| Non-asthmatic | M | 47 | 105 | 167.5 | Caucasian | CNS Tumor | N/A | None |

Supplementary Table S2. Differentially expressed genes ranked by p-value.

| **Gene Symbol** | **Gene Name** | **Counts ± SEM** | | **Fold Change** | **p-value (adjusted p-value)** |
| --- | --- | --- | --- | --- | --- |
|  |  | **Non-Asthmatic** | **Asthmatic** |  |  |
| ITGB6 | Integrin beta 6 | 475.3 ± 46.4 | 367.4 ± 27.9 | -1.29 | 0.000063 (0.002) |
| COL1A1 | Collagen, type I, alpha 1 | 735.8 ± 172.1 | 1344.4 ± 222.0 | 1.83 | 0.0003 (0.01) |
| COL3A1 | Collagen, type III, alpha 1 | 3321.1 ± 321.3 | 5324.3 ± 1517.9 | 1.60 | 0.001 (0.04) |
| RAC1 | Ras-related C3 botulinum toxin substrate 1 | 1383.3 ± 149.5 | 866.3 ± 74.1 | -1.60 | 0.002 (0.07) |
| CD38 | Cyclic ADP ribose hydrolase | 937.4 ± 74.0 | 562.9 ± 32.1 | -1.66 | 0.003 (0.10) |
| IL13RA1 | Interleukin 13 receptor, alpha 1 | 1868.0 ± 64.7 | 1428.3 ± 77.3 | -1.31 | 0.004 (0.11) |
| HSPB1 | Heat shock 27kDa protein 1 | 865.2 ± 61.9 | 1076.6 ± 136.5 | 1.24 | 0.004 (0.13) |
| PTGFR | Prostaglandin F receptor | 144.2 ± 18.3 | 66.1 ± 8.5 | -2.18 | 0.004 (0.13) |
| CALM2 | Calmodulin 2 | 6965.4 ± 856.4 | 3706.4 ± 292.2 | -1.88 | 0.005 (0.15) |
| ITGA3 | Integrin, alpha 3 | 861.6 ± 85.8 | 616.6 ± 38.4 | -1.40 | 0.006 (0.17) |
| ADAM33 | Disintegrin and metalloproteinase domain-containing protein 33 | 267.1 ± 37.9 | 416.8 ± 70.2 | 1.56 | 0.006 (0.18) |
| LAMC2 | Laminin, gamma 2 | 588.3 ± 76.0 | 369.1 ± 27.6 | -1.59 | 0.006 (0.19) |
| IFIH1 | Interferon induced with helicase C domain 1 | 163.5 ± 12.5 | 239.7 ± 30.2 | 1.45 | 0.006 (0.20) |
| IDO1 | Indoleamine 2,3-dioxygenase 1 | 125.2 ± 19.2 | 176.2 ± 34.4 | 1.41 | 0.007 (0.21) |
| NLRP3 | NACHT, LRR and PYD domains-containing protein 3 | 113.7 ± 16.9 | 179.5 ± 31.7 | 1.58 | 0.007 (0.21) |
| TGFBR2 | Transforming growth factor, beta receptor 2 | 2198.0 ± 121.0 | 1849.8 ± 110.2 | -1.19 | 0.01 (0.30) |
| RHOA | Ras homolog gene family, member A | 698.2 ± 82.4 | 493.2 ± 36.2 | -1.42 | 0.01 (0.34) |
| TJP1 | Tight junction protein 1 | 1258.1 ± 140.0 | 873.8 ± 81.8 | -1.44 | 0.01 (0.35) |
| CALM1 | Calmodulin 1 | 513.9 ± 66.2 | 228.1 ± 25.5 | -2.25 | 0.01 (0.35) |
| MMP8 | Matrix metalloproteinase-8 | 21.1 ± 3.2 | 59.1 ± 22.9 | 2.80 | 0.01 (0.40) |
| TGFB2 | Transforming growth factor, beta 2 | 88.5 ± 12.3 | 60.8 ± 7.9 | -1.46 | 0.01 (0.42) |
| SMTN | Smoothelin | 279.0 ± 25.5 | 390.6 ± 28.9 | 1.40 | 0.01 (0.45) |
| CAV2 | Caveolin 2 | 1075.6 ± 138.4 | 793.6 ± 104.0 | -1.36 | 0.01 (0.46) |
| PRKCE | Protein kinase C epsilon | 865.5 ± 45.9 | 681.6 ± 28.1 | -1.27 | 0.02 (0.47) |
| NFATC3 | Nuclear factor of activated T-cells, cytoplasmic, calcineurin-dependent 3 | 1478.1 ± 62.4 | 1185.6 ± 79.5 | -1.25 | 0.02 (0.56) |
| PPP1CC | Protein phosphatase 1, catalytic subunit, gamma | 668.1 ± 26.5 | 564.6 ± 15.5 | -1.18 | 0.02 (0.58) |
| SFTPD | Surfactant protein D | 1798.9 ± 146.7 | 1316.6 ± 189.0 | -1.37 | 0.02 (0.59) |
| BPIFA1 | BPI fold containing family A, member 1 | 387.3 ± 98.3 | 488.5 ± 188.3 | 1.26 | 0.02 (0.60) |
| LEPREL1 | Leprecan-like 1 | 431.9 ± 46.5 | 342.6 ± 40.8 | -1.26 | 0.02 (0.60) |
| LTB4R2 | Leukotriene B4 receptor 2 | 89.1 ± 3.9 | 102.7 ± 7.1 | 1.15 | 0.02 (0.62) |
| SUMF2 | Sulfatase modifying factor 2 | 1844.7 ± 137.7 | 1387.5 ± 101.7 | -1.33 | 0.02 (0.67) |
| WASL | Wiskott-Aldrich syndrome-like | 433.2 ±33.7 | 350.1 ± 23.8 | -1.24 | 0.02 (0.67) |
| FOS | FBJ murine osteosarcoma viral oncogene homolog | 1041.1 ± 127.4 | 1514.6 ± 198.2 | 1.45 | 0.02 (0.71) |
| PRMT5 | Protein arginine methyltransferase 5 | 402.7 ± 37.5 | 265.0 ± 7.9 | -1.52 | 0.03 (0.79) |
| AQP1 | Aquaporin 1 | 541.0 ± 35.2 | 468.5 ± 43.5 | -1.15 | 0.03 (0.86) |
| ITGAE | Integrin, alpha E | 107.7 ± 9.1 | 82.9 ± 2.4 | -1.30 | 0.03 (0.95) |
| ILK | Integrin-linked kinase | 738.0 ± 33.9 | 808.9 ± 30.3 | 1.10 | 0.03 (1.0) |
| CDH1 | Cadherin-1 | 943.2 ± 113.6 | 695.6 ± 56.4 | -1.36 | 0.03 (1.0) |
| DHX58 | DEXH (Asp-Glu-X-His) box polypeptide 58 | 209.4 ± 16.3 | 251.0 ± 15.7 | 1.20 | 0.03 (1.0) |
| AQP4 | Aquaporin 4 | 1409.3 ± 97.2 | 1644.6 ± 185.3 | 1.17 | 0.04 (1.0) |
| GNAQ | Guanine nucleotide binding protein (G protein), q polypeptide | 1449.7 ± 62.0 | 1336.3 ± 36.7 | -1.08 | 0.04 (1.0) |
| TLR7 | Toll-like receptor 7 | 30.6 ± 4.1 | 23.1 ± 1.7 | -1.32 | 0.04 (1.0) |
| BMP7 | Bone morphogenetic protein 7 | 27.6 ± 3.8 | 45.3 ± 5.1 | 1.64 | 0.04 (1.0) |
| COL7A1 | Collagen, type VII, alpha 1 | 111.5 ± 9.8 | 273.7 ± 47.9 | 2.45 | 0.04 (1.0) |
| RGS2 | Regulator of G-protein signaling 2 | 948.4 ± 108.5 | 1236.4 ± 117.9 | 1.30 | 0.04 (1.0) |
| ARHGEF11 | Rho guanine nucleotide exchange factor 11 | 157.8 ± 8.0 | 147.7 ± 6.9 | -1.07 | 0.04 (1.0) |
| F11R | F11 receptor | 1695.7 ± 118.0 | 1514.5 ± 115.1 | -1.12 | 0.04 (1.0) |
| TSLP | Thymic stromal lymphopoietin | 43.2 ± 8.4 | 19.4 ± 2.8 | -2.23 | 0.04 (1.0) |
| MMP7 | Matrix metalloproteinase-7 | 204.0 ± 24.8 | 85.0 ± 16.1 | -2.40 | 0.05 (1.0) |
| IL18RAP | Interleukin 18 receptor accessory protein | 421.3 ± 71.9 | 601.2 ± 91.7 | 1.43 | 0.05 (1.0) |
| MAPK1 | Mitogen-activated protein kinase 1 | 1133.1 ± 63.2 | 874.7 ± 40.6 | -1.29 | 0.05 (1.0) |

Table S3. Summary of genes differentially co-expressed in asthma or in non-asthma.

| Cluster # | Genes in Group | R in Non-asthmatics | p-value | R in Asthmatics | p-value |
| --- | --- | --- | --- | --- | --- |
| Co-Expression in Non-Asthmatics | | | | | |
| 10 | ALOX5, CD44, CHI3L1, COL1A1, FAM213B, GSDMB, HDAC10, IDO1, IFIH1, IL1B, IL1RN, KAT2A, LAMA4, NFATC1, NFATC2, NOTCH1, PRKX, PTGES, SELE, SMTN, STAT6, TBXA2R, TGFB1, THY1, ZYX | 0.7234 | <0.0001 | 0.1655 | 0.094 |
| 35 | IL18R1, PYCARD, SFTPA1, SFTPA2, STAT5A | 0.7450 | <0.0001 | 0.2898 | 0.126 |
| 53 | ITGAV, MMP1, NLRP3, PTGS2, S100A2, TPM3 | 0.8479 | <0.0001 | 0.2137 | 0.200 |
| Co-Expression in Asthmatics | | | | | |
| 6 | ADAM33, CCL20, KAT2A, MUC5B | 0.1509 | 0.117 | 0.6571 | <0.0001 |
| 11 | AQP1, BMP2, BSG, CDH5, GGT1, GNAS, NFATC2, PRKCQ, TBXA2R | 0.0869 | 0.321 | 0.6653 | <0.0001 |
| 16 | AURKA, BPIFA1, DDX58, DHX58, IDO1, IFIH1 | 0.1557 | 0.236 | 0.7559 | <0.0001 |
| 17 | BCL3, COL4A1, IL1B, IL1RN, NFATC1, PTGIR, SELE | 0.3383 | 0.073 | 0.8049 | <0.0001 |
| 20 | KIT, LAMB2, MAPK3, MMP9, PTGS1,PXN | 0.0822 | 0.343 | 0.7631 | <0.0001 |
| 29 | COL7A1, KRT19, KRT5, TPM2 | 0.2008 | 0.061 | 0.7236 | <0.0001 |

**Supplementary figure legends:**

Figure S1 - Sample images of IHC stains for A) CD38, B) FLNB, C) SMTN. Positive cells are noted with a dashed arrow. Asterisk labels ASM layer. Arrow points to epithelial area. L denotes lumen of each airway. Black scale bar represents 60μm.

Figure S2. Expression of A) CD38 in the epithelium of non-asthmatics (n=12), asthmatics (n=12). B) FLNB in the epithelium of non-asthmatics (n=12), asthmatics (n=12). C) SMTN in the ASM of non-asthmatics (n=11), asthmatics (n=11). * p<0.05. All values expressed as percent positive area ± SEM.

Figure S3. Non-asthmatic co-expression. X-axis: patient IDs, Y-axis: relative gene expression. Each blue line represents one genes expression values across patients.

Figure S4. Asthmatic Co-expression. X-axis: patient IDs, Y-axis: relative gene expression. Each blue line represents one genes expression values across patients.
